# Thyroid Hormone Status Interferes with Estrogen Target Gene Expression in Breast Cancer Samples in Menopausal Women

**DOI:** 10.1155/2014/317398

**Published:** 2014-02-20

**Authors:** Sandro José Conde, Renata de Azevedo Melo Luvizotto, Maria Teresa de Síbio, Célia Regina Nogueira

**Affiliations:** ^1^Department of Biological Science, São Paulo Federal Institute (IFSP), 18136-540 São Roque, SP, Brazil; ^2^Department of Internal Medicine, Division of Endocrinology and Metabolism, UNESP, 18618-000 Botucatu, SP, Brazil

## Abstract

We investigated thyroid hormone levels in menopausal BrC patients and verified the action of triiodothyronine on genes regulated by estrogen and by triiodothyronine itself in BrC tissues. We selected 15 postmenopausal BrC patients and a control group of 18 postmenopausal women without BrC. We measured serum TPO-AB, TSH, FT4, and estradiol, before and after surgery, and used immunohistochemistry to examine estrogen and progesterone receptors. BrC primary tissue cultures received the following treatments: ethanol, triiodothyronine, triiodothyronine plus 4-hydroxytamoxifen, 4-hydroxytamoxifen, estrogen, or estrogen plus 4-hydroxytamoxifen. Genes regulated by estrogen (TGFA, TGFB1, and PGR) and by triiodothyronine (TNFRSF9, BMP-6, and THRA) *in vitro* were evaluated. TSH levels in BrC patients did not differ from those of the control group (1.34 ± 0.60 versus 2.41 ± 1.10 **μ**U/mL), but FT4 levels of BrC patients were statistically higher than controls (1.78 ± 0.20 versus 0.95 ± 0.16 ng/dL). TGFA was upregulated and downregulated after estrogen and triiodothyronine treatment, respectively. Triiodothyronine increased PGR expression; however 4-hydroxytamoxifen did not block triiodothyronine action on PGR expression. 4-Hydroxytamoxifen, alone or associated with triiodothyronine, modulated gene expression of TNFRSF9, BMP-6, and THRA, similar to triiodothyronine treatment. Thus, our work highlights the importance of thyroid hormone status evaluation and its ability to interfere with estrogen target gene expression in BrC samples in menopausal women.

## 1. Introduction

For many years, associations between thyroid disorders and breast cancer (BrC) have raised questions regarding the involvement of thyroid hormone (TH) (either associated with estrogen receptor or not) on the development and progression of breast cancer, and significant research efforts have focused on this area [1–10]. Recently a study first showed that TH levels in postmenopausal women are positively related to BrC risk in a dose-response manner [9].

Prognostic and predictive factors are indispensable tools in neoplastic disease treatment [11], and estrogen receptor (ER) concentration is an important parameter in BrC prognosis [12]; ER status is an important consideration for BrC antiestrogen treatment [13]. Therefore, the presence and concentration of ER provide crucial information regarding tumors that respond to hormonal intervention [14]. Positive ER detection in BrC tissues is an indication of a tumor with hormonal dependence and indicates the benefit of endocrine therapy to this type of BrC [15]. Additionally, identification of positive or negative ER tumors can direct therapeutic strategies and clinical prognosis [16–18].

Given the known effect of TH on BrC, there is little information about how this hormone binds with the receptors of breast tumor cells. Previous research demonstrated TH receptors in the nuclei of MCF-7 cells [19], while other works confirmed the presence of TH receptors in BrC tissue, but without correlation with other hormonal receptors (ER or progesterone receptor) or tumor progression [20]. Shao et al. [21] showed that triiodothyronine (T3) potentiates estrogen action on ER-positive BrC cell lines.

We previously performed *in vitro* studies in MCF-7 and MDA-MB-231 cell lines to show that a supraphysiological concentration of T3 induces cell proliferation and expression of genes previously stimulated by estradiol (E2) independent of ER, with inhibition of T3 induction by tamoxifen (TAM) [7].

It is recognized that E2 and the hormonal status of a patient are important in BrC cell proliferation and treatment [15], and with respect to T3, while epidemiological studies have produced contradictory data regarding its effect on BrC [1, 22–27], laboratory studies have demonstrated its ability to induce BrC proliferation in a ER-dependent manner, possibly through crosstalk between the TH and E2 pathways [3, 7, 28].

We investigated thyroid hormones levels in menopausal BrC patients because of their lack of estrogen and verified the action of T3 on genes regulated by E2 (*TGFA*, *TGFB1,* and *PGR*) [7] and T3 (*TNFRSF9*, *BMP6,* and *THRA*) [29] in primary BrC tissues exhibiting the early stages of tumor progression.

## 2. Methods

### 2.1. Patients

The study was approved by the Cancer Hospital, Antônio Prudente Foundation, São Paulo, Brazil, and Ethics Committee, and all patients signed an informed consent form. Patients recruited to this study were newly diagnosed with breast cancer and underwent surgery at the Cancer Hospital, Antônio Prudente Foundation, São Paulo, Brazil. All cases were classified as tumor node metastasis stage I or II. Ages ranged from 48 to 55 years, and all patients were menopausal (amenorrhea for at least 1 year).

Patients were excluded for the following reasons: radio- or chemotherapy administration before surgery, hormone replacement therapy, any kind of previously diagnosed thyroid disease, chronic kidney failure, or recent elevation in serum creatinine values over those normally expected for that particular age. Other exclusion factors were abnormal hepatic function with aspartate aminotransferase, alanine aminotransferase, bilirubin, or alkaline phosphatase concentrations higher than twice the normal upper limit; use of *β*-blocking agents, aspirin, heparin, phenytoin, steroids, or dopamine in the month before or during the study; use of iodine-containing contrast agents in the six months before and during the study.

A control group consisted of 18 women aged 47 to 57 years whose recent mammograms indicated the absence of breast cancer. These mammograms were performed in the same week when anamnesis and blood samples were collected.

### 2.2. Immunohistochemistry

The presence of ER and progesterone receptors (PR) in tumors was determined by immunohistochemical staining using a monoclonal antibody to ER*α* (Upstate Biotechnology Inc., Lake Placid, NY, USA) and a monoclonal anti-PR antibody 636 (M3569, DakoCytomation). Biotinylated secondary antibodies (anti-mouse IgG or anti-rabbit IgG) were obtained from Vector Laboratories (Burlingame, CA, USA). Endogenous peroxidase in tissue sections was blocked by incubation with a solution of 1% hydrogen peroxide for 30 min, and antigen retrieval was performed by microwaving sections in 0.01 M citrate buffer (pH 6.0) for 20 min at 800 W. Antibodies were diluted individually in PBS containing 3% BSA. ER*α* antibody was used at a dilution of 1 : 500 and PR antibody was used at 1 : 100. Prior to addition of secondary antibody, tissue sections were rinsed in PBS containing 0.05% Tween 20. The reactions were developed with an avidin-biotin-peroxidase complex. Tumors known to be positive for the studied marker were considered to be positive controls. Tumors were considered positive with a moderate intensity of staining and the proportion of this intensity at more than 10% of cells [30].

### 2.3. Serum Dosage

Serum aliquots were analyzed for thyroid peroxidase antibody (TPOab), thyroid-stimulating hormone (TSH), free thyroxine (FT4), and E2 using commercially available kits (DPC, Los Angeles, CA, USA). The normal ranges were <35.0 UI/mL for anti-TPO negative, 0.4–4.0 *μ*U/mL for TSH, 0.8–2.0 ng/dL for FT4, and 0.0–30.0 pg/mL for E2 (after menopause).

### 2.4. Chemicals

E2, T3, and TAM were purchased from Sigma. Each was dissolved in ethanol to give a stock solution and used diluted for use in culture at the following concentrations: E2 at a physiological concentration of 10^−8 ^M, T3 at a supraphysiological concentration of 10^−8 ^M, and TAM at a pharmacological concentration of 10^−6 ^M [7].

### 2.5. Primary Culture

Fresh human breast carcinoma tissue remaining after pathological and prognostic analysis was obtained from collaborating surgeons and pathologists; it was trimmed free of fat and placed in phosphate-buffered saline. A Krumdieck tissue slicer (Alabama Research Corporation) was used to obtain 0.3 mm slices. Slices were divided into six 35 mm dishes and placed on siliconized lens paper floating in 2 mL organ culture medium. Each treatment was performed in a single dish containing 2 to 3 slices. Primary tissue was cultured in phenol-red-free RPMI supplemented with 10 U/mL penicillin, 100 *μ*g/mL streptomycin, 500 *μ*g/mL BSA, and 5 *μ*g/mL insulin (Life Technologies Corporation, Carlsbad, CA, USA). Dishes received the following treatments: dish 1: ethanol; dish 2: T3 (10^−8 ^M); dish 3: T3 (10^−8 ^M) plus TAM (10^−6 ^M); dish 4: TAM (10^−6 ^M); dish 5: E2 (10^−8 ^M); dish 6: E2 (10^−8 ^M) plus TAM (10^−6 ^M). Cultures were maintained at 37°C in a humidified atmosphere of 95% air/5% CO_2_. Medium changes were performed after 24 h and harvesting was performed after 48 h [31].

### 2.6. RNA Isolation and Reverse Transcription

Total RNA was extracted from slices by the guanidinium thiocyanate method and analyzed by electrophoresis using 1% agarose gels. One microgram of total RNA was reverse transcribed with SuperScript III First-Strand Synthesis System for RT-PCR (Invitrogen, no. 18080-051).

### 2.7. Measurement of Gene Expression by Quantitative Real-Time PCR

The real-time RT-PCR method with an Assay-on-Demand Gene Expression Product (Life Technologies, P/N 4331182) consisted of unlabeled PCR primers and a TaqMan MGB probe (FAM dye-labeled) optimized to work with the TaqMan Universal PCR Master Mix (P/N 4304437) in an ABI Prism 7700 system (PerkinElmer Life Sciences, Boston, MA, USA) and was employed to quantitatively measure transforming growth factor alpha (*TGFA; Hs00608187_m1*), transforming growth factor beta 1 (*TGFB1; Hs00998133_m1*), progesterone receptor (*PGR; Hs01556702_m1*), tumor necrosis factor receptor superfamily member 9 (*TNFRSF9; Hs00155512_m1*), bone morphogenetic protein 6 (*BMP6; Hs01099594_m1*), thyroid hormone receptors *α*/*β* (*THRA; Hs00268470_m1*, and *THRB; Hs00230861_m1*), and glyceraldehyde-3-phosphate dehydrogenase (*GAPDH; Hs02758991_g1*) mRNA expression (Applied Biosystems). All assays were performed in triplicate. The mRNA contents were normalized to GAPDH mRNA levels, and differences in expression were determined by the CT method described in the ABI user's manual (Life Technologies).

### 2.8. Statistical Analysis

Serum dosages were compared by nonparametric analysis of variance for the two-factor model (*P* < 0.05, Mann-Whitney test).

Gene expression comparisons were performed by the analysis of variance technique for an experiment with completely randomized blocks, complemented by the Tukey multiple comparison test for pairs of measurements, or the equivalent nonparametric procedure when data were not normally distributed. The level of significance was set at 95% (*P* < 0.05) for all presented data.

## 3. Results

### 3.1. Hormonal Status and Immunohistochemistry

Fresh tumor samples were used for ER and PR immunohistochemistry. Two samples were negative for ER, and six were negative for PR. ER-negative samples were excluded from gene expression analysis.

Serum determinations were performed prior to surgery. After surgery, new serum determinations were performed to confirm the previously obtained data. Three patients (numbers 01, 05, and 14) presented with clinical hyperthyroidism (low TSH with high FT4), while one patient (number 06) was positive for TPOab, and one patient (number 11) showed subclinical hypothyroidism (high TSH with normal FT4) (Table 1). There were no differences in E2 levels between controls and BrC patients. BrC patients presented with no significant differences in E2 and TSH levels compared with control patients. Mean serum values for thyroid hormone were statistically higher in BrC than control patients. TPOab was not observed in controls. FT4 levels were significantly higher in BrC patients than in controls (Table 2).

### 3.2. Primary Culture

All the tissues included in this study were positive for estrogen receptor and thyroid hormone receptor expression.

**Figure 1:**
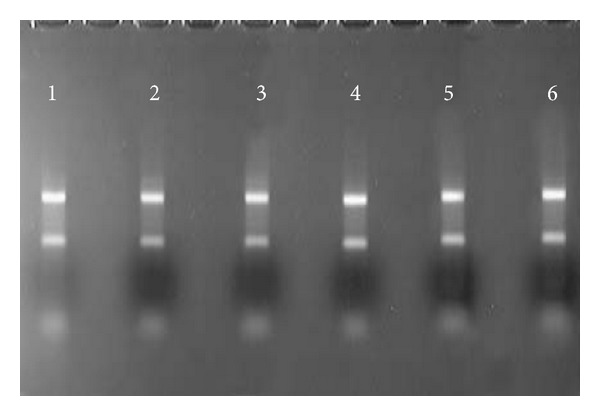
Total RNA from primary culture of breast tumor for different postsurgery treatment periods on a 1% agarose gel (1 : 8 h; 2 : 16 h; 3 : 24 h; 4 : 32 h; 5 : 40 h; 6 : 48 h).

To ascertain whether extracted mRNA quality was affected by treatment time, a 1% agarose gel test was used to evaluate intervals of 8, 16, 24, 32, 40, and 48 h (Figure 1). Of note, normal breast tissue from patients undergoing mammoplasty was negative for mRNA expression of the studied genes.

### 3.3. Gene Expression and Evidence of Thyroid Hormone Influence

**Figure 2:**
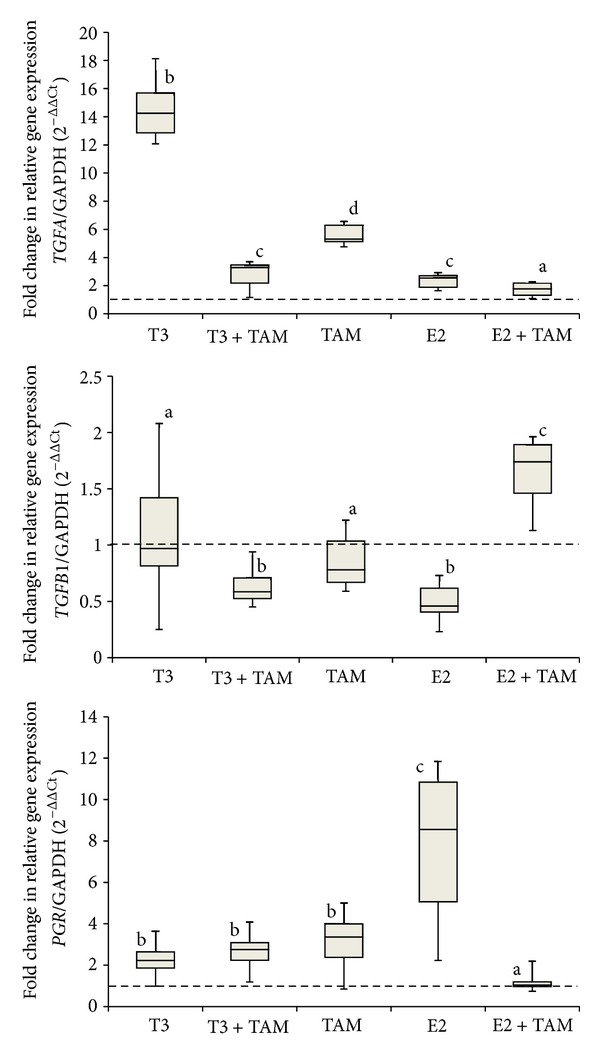
Gene expression of known estrogen-stimulated genes in primary cultures of breast tumors. Samples were treated as follows: ethanol vehicle, triiodothyronine (T3), T3 plus tamoxifen (TAM), TAM, estradiol (E2), and E2 plus TAM. After 48 h of treatment, *TGFA*, *TGFB1*, and *PGR* were quantified by real-time RT-PCR. Glyceraldehyde-3-phosphate dehydrogenase (*GAPDH*) was used to normalize gene expression. Relative mRNA expression was calculated using the expression level of the treated ethanol sample as the standard set to the dotted line and represented by letter “a.” Different letters indicate *P* < 0.05.

**Figure 3:**
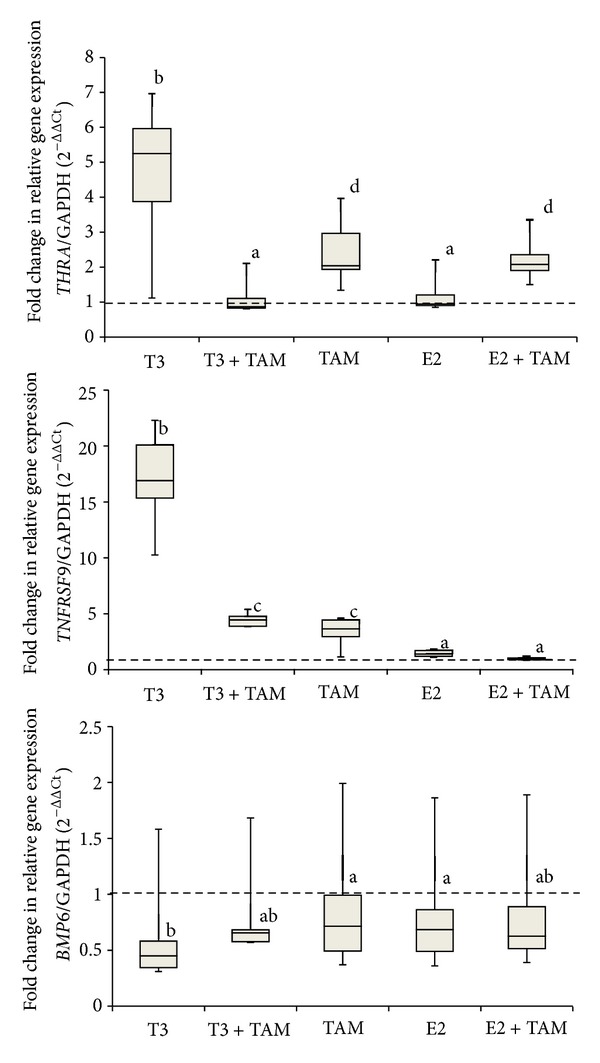
Gene expression of known thyroid hormone-stimulated genes in primary cultures of breast tumors. Samples were treated as follows: ethanol vehicle, triiodothyronine (T3), T3 plus tamoxifen (TAM), TAM, estradiol (E2), and E2 plus TAM. After 48 h of treatment, *THRA*, *TNFRSF9*, and *BMP6* were quantified by real-time RT-PCR. Glyceraldehyde-3-phosphate dehydrogenase (GAPDH) was used to normalize gene expression. Relative mRNA expression was calculated using the expression level in the treated ethanol sample as the standard set to the dotted line and represented by letter “a.” Different letters indicate *P* < 0.05.

Figure 2 shows box plots that represent the expression of each gene following stimulation by E2 (*TGFA, TGFB1,* and *PGR*) after administration of the previously described treatments; Figure 3 details the respective data for T3 (*TNFRSF9, BMP-6,* and *THRA*). Patients 01, 05, and 14 (with amended thyroid hormone status) and patients 03 and10 (negative ER) were excluded from these data.

## 4. Discussion

### 4.1. Hormonal Status and Immunohistochemistry

There is much evidence suggesting a relationship between thyroid disease and BrC risk but it is controversial. Numerous studies have been conducted, and some have found no significant relationship between thyroid disease [32] or treatment of thyroid disease [33] and BrC risk, while others correlated BrC with hyperthyroidism [1, 10, 34, 35], hypothyroidism [24, 36, 37], nontoxic goiter [8, 38, 39], or thyroid autoimmune diseases [8, 37, 38, 40–42].

With respect to thyroid diseases, previous studies of postmenopausal women have reported higher hypothyroidism incidence concurrent with BrC compared with women with other tumor types or normal controls [24]. These data were confirmed by studies on women with BrC independent of E2 status [35, 36, 42]. However, several studies were unable to confirm a relationship between hypothyroidism and BrC [39, 43]. Thyroid autoimmune disease is identified by TPOab positivity; several works identified higher TPOab in BrC patients than controls [8, 38, 41, 42]. Autoimmune cases as well as nontoxic goiters are common in BrC cases. Some works have shown significant increased thyroid volume in these cases [8, 42].

In our study, we identified a higher prevalence of thyroid disease (33.3%), with hyperthyroidism being the most frequent condition (20.0%), confirming previous reports [1, 27].

### 4.2. Primary Culture

One of the major concerns for researchers working with breast cancer models is that these models may exhibit behavior that is dissimilar to that of breast cancer tissue *in vivo*. Burdall and colleagues [44] evaluated the use of breast cancer cell cultures and highlighted the advantages of these models in that they exhibit limitless self-replication, are easily replaced with frozen stock, and demonstrate a relatively high degree of homogeneity. However, cell culture models are prone to genotypic and phenotypic drift, which excludes this model when considering and comparing individual characteristics of patients. A recent alternative approach has been aimed at minimizing the differences between *in vitro* culture models and living breast cancer tissue.

In contrast, culture of breast tumor slices does not present the effects of genetic drift, which may occur in cell lines during the course of passaging, and this method maintains some *in vivo* characteristics. Furthermore, breast tumor slices have been primarily useful for evaluating responses to hormonal and pharmacological treatments, showing gene expression results that are close to *in vivo* responses.

These data complement our understanding of how study models for breast cancer may present variations in results. It was previously observed by our group that TGFA expression in primary organ culture was not reproducible to the results seen in cell lines (5). Cell lines have advantages such as high genomic homogeneity that lead to little variation in the results when compared with primary organ cultures. However, it is now acknowledged that information regarding genomic variation is insignificant in comparison to the variability introduced during technical steps such as culture preparation and gene expression. In primary organ culture, variation in the data produced is higher than the natural variation introduced by the techniques used. These variations may reflect a heterogeneity that develops in different tumor samples because of the wide range of factors that lead to genomic instability.

### 4.3. Gene Expression and Evidence of Thyroid Hormone Influence

Some gene expression variation showing a marked action of E2 on breast cancer has already been established in previous works. We previously demonstrate upregulated TGFA and downregulated TGFB1 after E2 and T3 treatments. We reported this variation in breast cancer cells lines, with estrogen receptor dependence [7]. When hormonal treatment was associated with TAM, variations in gene expression did not show statistical differences compared with control cells. However, although E2 and T3 treatment of primary cultures showed the same gene expression profile, TAM association did not block T3 treatment but rather increased TGFA and decreased TGFB1 gene expression during T3 and TAM association. Here, we choose PGR as a marker of estrogen action, as reported elsewhere [45]. As expected, E2 treatment increased PGR expression and TAM association blocked this action. Although T3 treatment increased PGR expression, this action was less pronounced than E2; however, TAM did not block T3 action on PGR expression, diverging from data previously obtained from cell lines [7]. Treatment with TAM can inhibit E2 action, functioning like a partial antagonist of nuclear ER, but there is evidence for the agonistic action of TAM on plasma membrane receptors for E2 [46]. Our group has previously correlated PGR expression with TH [7], and here we provided evidence for the upregulation of PGR in primary culture treated with T3 plus TAM.

To confirm the action of T3 treatment, we verified the expression of previously described genes exhibiting upregulation (TNFRSF9 and THRA) or downregulation (4-1BB) under TH treatment [29]. Treatment of primary cultures with T3 and E2 reproduced equivalent results obtained in cell lines [7]. However, associations of these hormones with TAM did not reproduce these results. Acting as antagonist on ER, TAM associated with E2 maintained expression of TGFA and PGR to the control levels, but when T3 plus TAM association was applied, TGFA and TGFB1 expression was at the same levels as those following E2 treatment. This association may coincide with estrogenic response on gene expression and highlight the effect of TAM usage in hyperthyroid patients, especially considering the proliferative and apoptotic effects of TGFA and TGFB1, respectively, on the initial tumor progression stages. This finding was reinforced by observations of upregulated PGR expression under T3 treatment (although levels were less than those produced following E2 stimulation), which did not show changes with TAM association.

To assess whether E2 could influence the expression of T3 target genes, we examined the effect on TNFRSF9, BMP-6, and THRA expression and observed no differences in comparison to the control. E2 and TAM treatment upregulated THRA expression; however, this effect may be due only to TAM because it exerted the same upregulation effects as E2 plus TAM.

Note that TAM alone or associated with T3 modulated gene expression of TNFRSF9, BMP-6, and THRA related to the control, similar to T3 treatment, showing that the TAM can interfere with gene expression modulated by T3.

Our results showed that thyroid dysfunctions correlate with ER positivity (Table 1). There are many possibilities of crosstalk between T3, E2, and TAM. Some previous research considered TH and ER binding, as demonstrated in BrC cell lines [7]. On the other hand, TH can alter ER-dependent gene transcription, with ER-thyroid hormone receptor dimer formation that results in flexible regulation of the consensus ERE [28], or interaction between thyroid hormone receptors and ERE [3]. Most recently, studies have emphasized the nongenomic actions of T3 and E2, commencing at the receptors at the plasma membrane or in the cytoplasm and activating intracellular signaling pathways such as PI3 K or MAPK. The E2 antagonist fulvestrant does not activate these signaling pathways by this mechanism, but other selective estrogen receptor modulators (SERM), such as TAM, do it in a similar manner to E2, showing that TAM confers an antagonistic action on nuclear ER but an agonistic action on membrane ER [47, 48]. AKT and MAPK phosphorylation can elevate agonistic TAM and other SERM activity [48].

## 5. Conclusions

Our work identified a high incidence of hyperthyroidism in menopausal women with BrC, who exhibited a higher degree of TH than the control group. Primary culture of tumor samples was utilized to evaluate gene expression modified by T3 or E2 treatment and produced similar but not identical results to those observed in breast cancer cell lines. T3 had a significant effect on genes classically regulated by E2, but the combination of T3 with TAM did not reverse gene expression levels to those observed in the untreated control group, in contrast to E2 plus TAM, which resulted in maintained gene expression when E2 treatment was applied. Taking into account the known ability of TH to mimic the effects of E2, particularly in the presence of TAM, our results reinforce previous reports that the thyroid hormone status of BrC patients can influence E2-controlled mechanisms, even under TAM intervention and/or the absence of circulating E2 in postmenopausal women. Thus, our study highlights the importance of evaluation of thyroid hormone status when considering the prognosis and treatment options for individual patients.

## Figures and Tables

**Table 1:** Tumor staging, immunohistochemistry, and serum dosage of breast cancer patients.

Case number	Staging	Immunohistochemistry	Serum dosage			
			TPOAb^(1)^ (UI/mL)	TSH^(2)^ (*μ*I/mL)	FT4^(3)^ (ng/dL)	E2^(4)^ (pg/mL)
1^a^	T(2) N(1) M(0)	ER(+); PR(+)	11.50	0.03	3.38	<20.00
2	T(2) N(0) M(0)	ER(+); PR(−)	<10.00	3.03	1.48	26.70
3	T(2) N(0) M(0)	ER(−); PR(−)	22.30	1.22	1.78	<20.00
4	T(1) N(0) M(0)	ER(+); PR(+)	22.90	0.96	1.44	26.80
5^a^	T(2) N(0) M(0)	ER(+); PR(+)	<10.00	0.30	1.94	<20.00
6^b,c^	T(1) N(2) M(0)	ER(+); PR(+)	354.00	5.11	1.26	<20.00
7	T(2) N(1) M(0)	ER(+); PR(−)	<10.00	1.85	2.30	26.90
8	T(2) N(0) M(0)	ER(+); PR(−)	23.90	1.83	1.75	26.10
9	T(1) N(0) M(0)	ER(+); PR(+)	13.50	1.34	1.85	28.20
10	T(2) N(1) M(0)	ER(−); PR(+)	18.40	0.92	1.85	23.80
11^b^	T(2) N(0) M(0)	ER(+); PR(+)	<10.00	4.47	1.39	<20.00
12	T(2) N(0) M(0)	ER(+); PR(−)	<10.00	1.60	1.53	<20.00
13	T(2) N(1) M(0)	ER(+); PR(+)	11.90	1.80	1.77	<20.00
14^a^	T(1) N(0) M(0)	ER(+); PR(−)	<10.00	0.29	3.06	26.70
15	T(2) N(0) M(0)	ER(+); PR(+)	15.70	0.76	1.78	24.50

^(1)^Thyroid peroxidase antibody (TPOab): <35.00 UI/mL = negative.

^(2)^Thyroid-stimulating hormone (TSH): normality between 0.4 and 4.0 mUI/mL.

^(3)^Free thyroxine (FT4): normality between 0.8 and 1.9 ng/dL.

^(4)^Estradiol (E2): normality on postmenopause between 0 and 30 pg/mL.

^a^Hyperthyroidism.

^b^Subclinical hypothyroidism.

^c^TPOab positive.

**Table 2:** Comparison of hormonal dosages between breast cancer and normal control patients.

	Breast cancer (*N* = 15)	Control (*N* = 18)
TSH (*μ*I/mL)	1.34 ± 0.60	2.41 ± 1.10
FT4 (ng/dL)	1.78 ± 0.20*	0.95 ± 0.16
E2 (pg/mL)	23.80 ± 3.35	21.80 ± 3.29

Data are reported as median ± total semirange. TSH: thyroid-stimulating hormone; FT4: free thyroxine; E2: estradiol. *P < 0.05 compared with control group (Mann-Whitney test).

## Abbreviations

BrC: Breast cancer
TH: Thyroid hormone
ER: Estrogen receptor
T3: Triiodothyronine
E2: Estradiol
TAM: 4-Hydroxytamoxifen
PR: Progesterone receptor
TPOab: Thyroid peroxidase antibody
TSH: Thyroid-stimulating hormone
FT4: Free thyroxine
TGFA: Transforming growth factor alpha
TGFB1: Transforming growth factor beta 1
PGR: Progesterone receptor
TNFRSF9: Tumor necrosis factor receptor superfamily 9
BMP-6: Bone morphogenetic protein 6
THRA: Thyroid hormone receptor alpha.
